# Central Venous Catheter-related Thrombosis in a Dialysis Patient: A Case Report

**DOI:** 10.31729/jnma.7603

**Published:** 2022-07-31

**Authors:** Sachit Regmi, Dilasha Manandhar, Sulav Pyakurel, Bibek Shrestha, Pitamber Khanal, Sandip Paudel, Pawan Gyawali

**Affiliations:** 1 Nepal Mediciti Hospital, Karyabinayak, Lalitpur, Nepal; 2 Nepal Medical College and Teaching Hospital, Jorpati, Kathmandu, Nepal; 3 Om Hospital and Research Center, Pvt. Ltd., Chabahil, Kathmandu, Nepal; 4 Patan Academy of Health Sciences, Lagankhel, Lalitpur, Nepal; 5 Tribhuvan University Teaching Hospital, Maharajgunj, Kathmandu, Nepal

**Keywords:** *central venous catheterization*, *chronic kidney disease*, *hemodialysis*, *thrombus*

## Abstract

Hemodialysis is one of the treatment modalities for advanced kidney disease and can help an individual live an active life despite failing kidneys. Although it improves the quality of life, it is not completely risk-free. It has several complications, among which, thrombus formation is common. We report a case of a 63-year-old man who presented at our institution for regular hemodialysis with recurrent arteriovenous graft failure. Because doppler ultrasound is a non-invasive procedure that can identify a thrombus in a vein, it is the best initial option for patients with internal jugular vein thrombosis. The use of ultrasound not only can guide a catheter pathway but can also help in early diagnosis and prevent complications following catheterization in a vein with a thrombus.

## INTRODUCTION

Central venous catheterization is an option used for emergency hemodialysis. Temporary dialysis catheter for hemodialysis inserted in the internal jugular vein, which is preferred, has various early and late complications despite being relatively unchallenging for bedside insertion. One such complication is venous thromboembolism, and catheter insertion in a vein with a thrombus may lead to fatal outcomes like pulmonary embolism, septic emboli and brain oedema. In a prospective study, the mortality rate of internal jugular vein thrombosis was reported to be 44%.^1^ This rate is significantly higher in older patients with underlying comorbidities such as malignancy, chronic kidney diseases and infections.^2^

## CASE REPORT

A 63 year-old-male who was a known case of chronic kidney disease (CKD) and under maintenance hemodialysis twice a week for 1 year presented to our institute for his regular hemodialysis with complaints of shortness of breath for 1 day, decreased urine output, generalised weakness and whole body generalised oedema since few days. He had no history of any neck trauma, intravenous drug abuse, fever, mass or neck swelling. He was referred to the intensive care unit for emergency dialysis after the failure of his regular dialysis within 30 minutes. He was also a known case of hypertension and diabetes mellitus type 2 under medication for 6 years. While taking history he gave a history of multiple access failures. He had no known personal or family history of clotting disorders, deep venous thrombosis (DVT) or hypercoagulable states. The history of the first emergency right Internal Jugular Vein catheterization for dialysis was around 1 year back. Later a fistula was created in the left hand which got thrombosed and stopped functioning around 6 months back. Consequently, the internal jugular vein (IJV) was catheterized to continue his maintenance hemodialysis (HD) which eventually showed signs of infection just after a month. The flow also started decreasing in his left IJV and finally stopped working after a while. This led to right IJV catheterization but unfortunately flow started to decrease as well after a few weeks and his maintenance dialysis could not be completed due to access failure.

**Figure 1:**
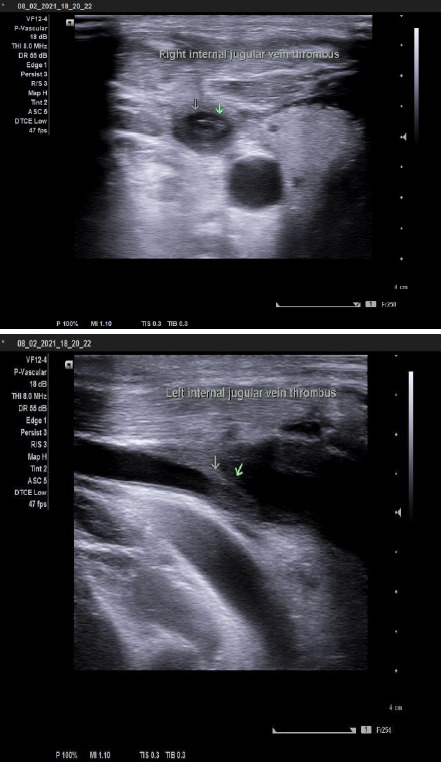
Thrombus in the bilateral internal jugular vein.

Investigations did reveal anaemia with a hematocrit of 30.7% and a normocytic normochromic picture. WBC count was 25400/cumm with neutrophil 85% and platelet count was 251000/cumm. His prothrombin time was 19.1 seconds and international normalized ratio (INR) 1.55. Serum urea and creatinine were 172.1 mg/dl and 12.3 mg/dl respectively. Serum Na^+^ was 122 meq/l, K+ 6.8 meq/l and phosphorus 9.7 mg/ dl. Activated partial thromboplastin time (APTT) was 140.9 second. His arterial blood gas analysis was normal. Ultrasonography (USG) screening of the neck was done which revealed bilateral (B/L) IJV thrombosis, hence dialysis catheter was placed with USG guidance on the right femoral vein with a plan of peritoneal dialysis catheter insertion (Figure 1).

Color Doppler study of neck vessels was performed which revealed an absence of spectrum Doppler signal in distal one third of B/L IJV. Vein contained a significant amount of partially occluding thrombus with a reduced flow pattern and was not compressible with other age-related atherosclerotic changes seen in b/l common carotid arteries (CCA) and internal carotid arteries with few small calcified plaques in b/l carotid bulbs and CCA.

## DISCUSSION

Venous thromboembolism is predisposed in CKD due to underlying hemostatic derangements like activation of procoagulant markers, decreased endogenous anticoagulants, enhanced platelet activation and aggregation and decreased activity of a fibrinolytic system.^3^ We do not have many high-quality studies related to CKD and based on limited studies and limited available data CKD affects around 10% of the population and the risk increases with age.^4^

The development of thrombosis may also be related to the duration of the indwelling catheter.^5^ A published study reported IJV thrombosis in a quarter of HD patients within 10 days of catheterization with the rate increasing progressively after the time of catheterization.^2,5^

Catheter-related DVT occurs more commonly than is reported. In a prospective study, it was associated with 26% of inserted central venous catheters.^2^ Up to 70% of the DVT-catheter-related thrombosis was reported with an IJV catheterization compared with 30% for the subclavian vein (SCV).^6^

Pulmonary embolism is the most common complication in upper extremity thrombosis, followed by post-thrombotic syndrome and death.^7^ Complications of IJV thrombosis are pulmonary embolism (10.3%) and post-thrombotic syndrome (41.4%).^2^ In Lemierre's syndrome, without proper antibiotic management, 97% of cases developed septic emboli in the lung.^8^

Doppler ultrasound is the best initial option for patients with IJV thrombosis because it is non-invasive and reliable as well.^9^ We can see a hyperechoic mass within the IJV. The use of ultrasound guidance during catheterization has however not shown any effect on the risk of catheter-related thrombosis, but it can help in early diagnosis and prevent the complications following catheterization in vein with thrombus. Although venography is the gold standard for diagnosing, there are risks of contrast and radiation exposure so it should only be done in patients with high clinical suspicion.^10^ Where there are facilities, magnetic resonance imaging (MRI), and nuclear medicine scanning can also be done.

Minimizing the use of central venous catheters for HD remains the best approach to preventing adverse complications such as thrombosis.^11^ According to the National Kidney Foundation-Kidney Disease Quality Initiative (NKF-KDOQI), AVF remains preferred among ESRD patients receiving HD.^12^

Because there are few evidence-based guidelines for treating central venous catheter-related thrombosis, it is always better to avoid the worst-case scenario.

Furthermore, when utilizing such catheters in unavoidable circumstances, rigorous planning and care, as well as watchful surveillance to detect difficulties early, will help to reduce related morbidity. One excellent modality is the use of ultrasound for screening catheter related thrombosis.

## References

[1] Drakos P, Ford BC, Labropoulos N (2020). A systematic review on internal jugular vein thrombosis and pulmonary embolism.. J Vasc Surg Venous Lymphat Disord..

[2] Lee Y, Siddiqui WJ (2021). StatPearls [Internet].

[3] Wattanakit K, Cushman M (2009). Chronic kidney disease and venous thromboembolism: epidemiology and mechanisms.. Curr Opin Pulm Med..

[4] Coresh J, Selvin E, Stevens LA, Manzi J, Kusek JW, Eggers P (2007). Prevalence of chronic kidney disease in the United States.. JAMA..

[5] Yardim H, Erkoc R, Soyoral YU, Begenik H, Avcu S (2012). Assessment of internal jugular vein thrombosis due to central venous catheter in hemodialysis patients: a retrospective and prospective serial evaluation with ultrasonography.. Clin Appl Thromb Hemost..

[6] Trerotola SO, Kuhn-Fulton J, Johnson MS, Shah H, Ambrosius WT, Kneebone PH (2000). Tunneled infusion catheters: increased incidence of symptomatic venous thrombosis after subclavian versus internal jugular venous access.. Radiology..

[7] Grigorian A, Nahmias JT (2022). StatPearls [Internet].

[8] Chaker K, Berrada O, Lyoubi M, Oukessou Y, Abada RA, Rouadi S (2021). Lemierre's syndrome or re-emerging disease: Case report and literature review.. Int J Surg Case Rep..

[9] Mohammad Shameem M, Akhtar A, Bhargava R, Ahmed Z, Baneen U, Khan NA (2010). Internal jugular vein thrombosis - A rare presentation of mediastinal lymphoma.. Respiratory Medicine CME..

[10] Karande GY, Hedgire SS, Sanchez Y, Baliyan V, Mishra V, Ganguli S (2016). Advanced imaging in acute and chronic deep vein thrombosis.. Cardiovasc Diagn Ther..

[11] Gunawansa N, Sudusinghe DH, Wijayaratne DR (2018). Hemodialysis Catheter-Related Central Venous Thrombosis: Clinical Approach to Evaluation and Management.. Ann Vasc Surg..

[12] National Kidney Foundation. (2002). K/DOQI clinical practice guidelines for chronic kidney disease: evaluation, classification, and stratification.. Am J Kidney Dis..

